# The Effectiveness of Real-Time Feedback with an Audible Pulse: A Preliminary Study in Renal Doppler Ultrasonography

**DOI:** 10.1371/journal.pone.0163953

**Published:** 2016-09-29

**Authors:** Min Hee Lee, Hae Kyung Lee, Seo-Youn Choi, Boem Ha Yi

**Affiliations:** Department of Radiology, Soonchunhyang University Bucheon Hospital, Bucheon-si, Gyeonggi-do, South Korea; Shenzhen Institutes of Advanced Technology, CHINA

## Abstract

**Purpose:**

The effectiveness of real-time feedback using an audible pulse in renal Doppler ultrasonography was evaluated.

**Methods:**

This study was approved by the institutional review board of our hospital. Written informed consent was provided by all volunteers at enrollment. The 26 healthy volunteers enrolled in this study underwent Doppler ultrasound of both kidneys using audible and inaudible pulses in randomized order and at 1-week intervals. Doppler waveforms were obtained at the interlobar or arcuate arteries using a 2-mm Doppler gate. Each session was considered complete when reproducible waveforms were obtained for 5 s in three predefined regions of the kidney. The scan times needed to obtain waveforms of the right and left kidneys were recorded separately. Measurements were compared using a paired *t*-test and a two-sample Wilcoxon rank-sum test.

**Results:**

The total recorded Doppler sonography scan time for each kidney ranged from 33 to 146 s. The mean scan time was 56.83 s (right, 58.19 s; left, 55.46 s) in the audible session and 72.58 s (right, 72.08 s; left, 73.08 s) in the inaudible session. The scan times were significantly shorter in the audible than inaudible session (p<0.001), whereas the difference in the scan times between the right and left kidneys was not significant. The order of the sessions had no effect on the total scan time.

**Conclusion:**

Real-time feedback using an audible pulse may encourage patient cooperation during breath-holding and can shorten the time needed to perform Doppler ultrasonography.

## Introduction

In renal Doppler ultrasonography, the resistive index (RI), defined as the (peak systolic velocity—end diastolic velocity)/peak systolic velocity at the renal segmental arteries, is considered to be a good indicator of renal vascular resistance [1–3]. In addition to being noninvasive, previous reports have shown that RI measurements provide useful diagnostic information regarding various renal diseases, including type 2 diabetes [3–6]. However, even minimal changes in respiration or position can influence the Doppler waveforms and thus the successful measurement of RI [7]. Consequently, both the time needed for Doppler assessment and the quality of the scan are largely dependent on the ability of the patient to hold his or her breath for the required duration.

We recently observed that cooperation during Doppler ultrasonography is better when the patient can hear the sound of the pulses. Thus, the purpose of this study was to prospectively determine the clinical effectiveness of real-time feedback using audible pulses in renal Doppler ultrasonography.

## Materials and Methods

This study was approved by the Institutional Review Board of Soonchunhyang University Bucheon Hospital. Written informed consent was provided by all participants at enrollment.

### Study Population

From February to March 2012, healthy volunteers were enrolled using the following inclusion criteria: (a) aged 20–55 years, (b) absence of renal symptoms or known renal disease, and (c) no history of dyspnea. We recruited volunteers who responded to personal solicitation and agreed to participate in the study. The risks and benefits of participation were discussed with each volunteer as part of the informed consent process, as required by our hospital’s institutional review board. Volunteers received fee-free abdominopelvic ultrasonography in exchange for their participation in the study.

### Imaging and Interpretation

Doppler sonography was performed using a LOGIQ E9 scanner (GE Healthcare, Waukesha, WI, USA) with a 1- to 5-MHz convex probe. The operator was an abdominal radiologist with 2 years of teaching experience.

Sonography consisted of two sessions using audible and inaudible pulses. Using the LOGIQ E9, the association between the sound volume and the gain of the Doppler wave can be uncoupled. Thus, during the monitoring of waveforms of the same intensity, the volume could be lowered to zero during the inaudible sessions and made sufficiently audible during the audible sessions. This allowed the radiologist to ask that the volunteers wait for the sound, without any loss of Doppler gain.

For this randomized cross-over study, all volunteers participated in both the audible and inaudible sessions at 1-week intervals. Except for the 1-week interval between sessions, the order of audible and inaudible sessions was arranged randomly to avoid learning or memory bias in the volunteers.

The right and left kidneys of all volunteers were evaluated in each session, and the scan time to obtain waveforms for each one was recorded separately. Doppler sonography was performed at the upper pole, the interpolar region, and at the lower pole of each kidney. Waveforms were obtained at the segmental arteries using a 2-mm Doppler gate. They were optimized for measurement using the lowest pulse repetition frequency without aliasing, the highest gain without obscuring flow by surrounding background noise, and the lowest wall filter.

**Fig 1:**
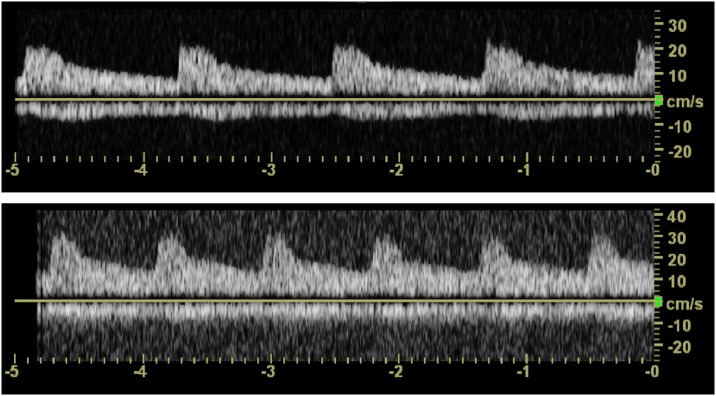
Full frame of uniform waves for 5 seconds. The Numbers of waves are different by the heart rates of participants (A, 48 beats per minute; B, 72 beats per minute).

Each session was considered complete when reproducible waveforms were obtained for 5 s in each of the three regions. Since variations in heart rate can affect the time needed for sonography, a full frame of uniform waves for 5 seconds was obtained, rather than a fixed number of waves (Fig 1). If within those 5 seconds the waveforms changed because of respiration or movement by the participants, a new waveform was obtained.

### Statistical Analysis

Statistical analysis was performed using commercial software (STATA, release 11; Stata Corp, College Station, TX, USA). A paired t-test was used to compare the duration of the audible and inaudible sessions and the scan times needed for the right and left kidneys. Differences in the order of the sessions were evaluated using the two-sample Wilcoxon rank-sum test. Linear regression analysis was performed to detect potential positive correlations between the volunteers’ body mass index (BMI) and the scan time.

## Results

The 9 male and 17 female volunteers enrolled in the study had a mean age of 39.3 ± 6.76 years. The mean BMI was 23.16 ± 3.48 kg/m^2^.

**Fig 2:**
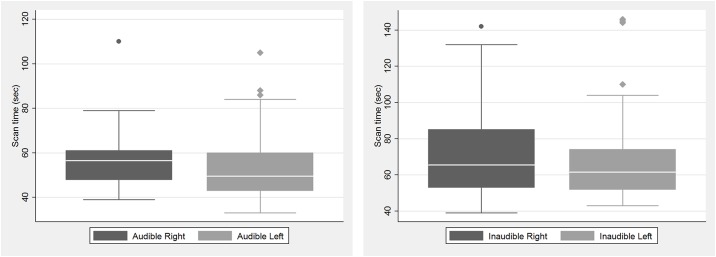
Scan time of right and left kidney. Box-and-whisker plots show Doppler sonographic scan time of right and left kidney during each session. There are no significant differences in the scan times between the right and left kidneys during the audible (A) and inaudible sessions (B).

**Fig 3:**
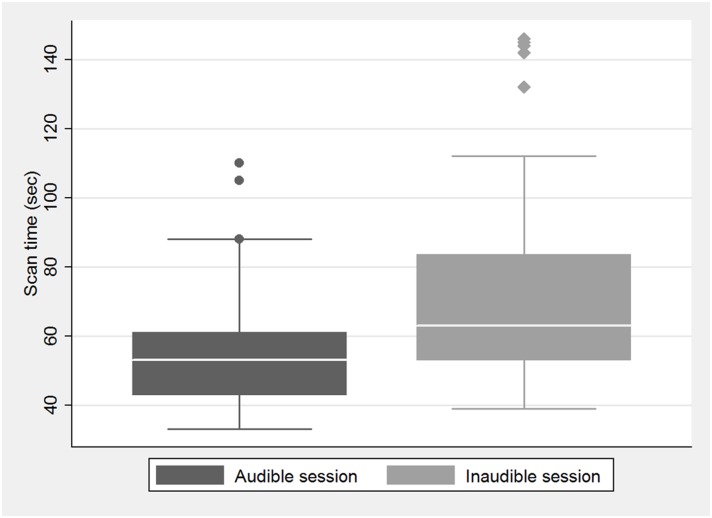
Scan time of audible and inaudible session. Box-and-whisker plots show scan time of the audible and inaudible sessions of all 52 kidneys, the time needed to obtain reproducible waveforms is significantly shorter during the audible than the inaudible sessions (p<0.001).

The total recorded Doppler sonography scan time for each kidney ranged from 33 to 146 s. In the audible session, the mean time was 56.83 s (right, 58.19 s; left, 55.46 s), and in the inaudible session, it was 72.58 s (right, 70.08 s; left, 73.08 s). There were no significant differences in the scan times between the right and left kidneys during both sessions (Fig 2). Among the 26 volunteers, 13 were examined first during an audible and then during an inaudible session 1 week later. The other 13 volunteers underwent the two sessions in the reverse order. However, the order of the sessions had no effect on the scan time. A paired t-test was used to compare the scan time of the audible and inaudible sessions of all 52 kidneys, the time needed to obtain reproducible waveforms was significantly shorter during the audible than the inaudible sessions (p<0.001) (Fig 3). There was no relationship between sonography scan time and the BMI of the volunteers.

## Discussion

Renal Doppler sonography was first introduced in the 1980s for the screening of patients with renovascular disease. Since then, many studies have reported the potential of Doppler sonographic assessments in this setting [7]. Mancini et al. found that diabetic patients with higher RI values had a higher degree of proteinuria, which suggests the value of this measurement in the early diagnosis of diabetic nephropathy [6].

In our institution, we routinely measure renal RI in diabetes mellitus patients as a means to predict diabetic nephropathy [8]. To obtain an adequate measurement of RI, patient breath-holding is necessary. However, this can be difficult, since most patients are unaware of the significance of breath-holding during the examination and how it is achieved. Thus, patients may move their abdomen or breathe unconsciously, despite attempts at breath-holding. We recently observed that cooperation is better when patients can hear the sound of the pulses during Doppler ultrasonography. Because the sound provides feedback, at the sound of the pulse, the patient can try to maintain its uniformity, which is a more concrete and specific task than breath-holding. The results of this study showed that RIs can be obtained much faster when volunteers are able to hear the pulse sound. We think that the shortened scan time reflects an improvement in efficiency of the renal Doppler ultrasonography.

Our study had several limitations. First, because all examinations were performed by a single radiologist, interobserver variability was not assessed. Second, for obvious reasons, the radiologist could not be “blinded” as to whether each session was an audible or inaudible one. This is a potential source of bias and may have been more likely to shorten the scan time in the audible session. Third, the study population was limited to healthy volunteers, and the effect of an audible pulse on RI measurements in elderly or obese patients was not determined. There will be the need for further study in more challenging individuals to establish the utility of these measurements.

Nonetheless, our study supports the use of real-time feedback using an audible pulse to encourage patient cooperation during breath-holding, as it can shorten the time needed to perform Doppler ultrasonography.

## Supporting Information

S1 Table 26 Volunteers’ Clinical Dataset. (DOCX)

## References

[1] Buscemi S, Verga S, Batsis JA, Cottone S, Mattina A, Re A, et al Intra-renal hemodynamics and carotid intima-media thickness in the metabolic syndrome. Diabetes research and clinical practice. 2009;86(3):177–85. 10.1016/j.diabres.2009.09.015 . 19815301

[2] Florczak E, Januszewicz M, Januszewicz A, Prejbisz A, Kaczmarska M, Michalowska I, et al Relationship between renal resistive index and early target organ damage in patients with never-treated essential hypertension. Blood Press. 2009;18(1–2):55–61. 10.1080/08037050902864078 . 19353412

[3] Raff U, Schmidt BM, Schwab J, Schwarz TK, Achenbach S, Bar I, et al Renal resistive index in addition to low-grade albuminuria complements screening for target organ damage in therapy-resistant hypertension. Journal of hypertension. 2010;28(3):608–14. 10.1097/HJH.0b013e32833487b8 . 20090556

[4] Radermacher J, Mengel M, Ellis S, Stuht S, Hiss M, Schwarz A, et al The renal arterial resistance index and renal allograft survival. The New England journal of medicine. 2003;349(2):115–24. 10.1056/NEJMoa022602 . 12853584

[5] Radermacher J, Chavan A, Bleck J, Vitzthum A, Stoess B, Gebel MJ, et al Use of Doppler ultrasonography to predict the outcome of therapy for renal-artery stenosis. The New England journal of medicine. 2001;344(6):410–7. 10.1056/NEJM200102083440603 . 11172177

[6] Mancini M, Masulli M, Liuzzi R, Mainenti PP, Ragucci M, Maurea S, et al Renal duplex sonographic evaluation of type 2 diabetic patients. Journal of ultrasound in medicine: official journal of the American Institute of Ultrasound in Medicine. 2013;32(6):1033–40. 10.7863/ultra.32.6.1033 . 23716525

[7] Taori KB, Chaudhary RS, Attarde V, Dhakate S, Sheorain V, Nimbalkar P, et al Renal Doppler indices in sickle cell disease: early radiologic predictors of renovascular changes. AJR American journal of roentgenology. 2008;191(1):239–42. 10.2214/AJR.07.3125 . 18562752

[8] Ohta Y, Fujii K, Arima H, Matsumura K, Tsuchihashi T, Tokumoto M, et al Increased renal resistive index in atherosclerosis and diabetic nephropathy assessed by Doppler sonography. Journal of hypertension. 2005;23(10):1905–11. 10.1097/01.hjh.0000181323.44162.01 . 16148615

