# Supplementary material for: The Effectiveness of Real-Time Feedback with an Audible Pulse: A Preliminary Study in Renal Doppler Ultrasonography
Source: PLoS One. 2016 Sep 29;11(9):e0163953. doi: 10.1371/journal.pone.0163953 (PMC5042422; doi:10.1371/journal.pone.0163953)
Supplement: S1 Table — (DOCX) [file pone.0163953.s001.docx]

| No. | Sex | Age | Weight (kg) | Height (m) | BMI | Initial Session | Audible session, right scan time (s) | Audible session, left scan time (s) | Inaudible session, right scan time (s) | Inaudible session, left scan time (s) |
| --- | --- | --- | --- | --- | --- | --- | --- | --- | --- | --- |
| 1 | F | 49 | 46 | 1.57 | 18.66 | Audible | 110 | 74 | 112 | 146 |
| 2 | F | 32 | 49 | 1.56 | 20.13 | Inaudible | 46 | 69 | 93 | 144 |
| 3 | F | 52 | 55 | 1.60 | 21.48 | Audible | 79 | 105 | 78 | 104 |
| 4 | F | 54 | 56 | 1.64 | 20.82 | Inaudible | 55 | 59 | 87 | 145 |
| 5 | F | 39 | 64 | 1.67 | 22.95 | Audible | 79 | 49 | 83 | 54 |
| 6 | F | 34 | 50 | 1.63 | 18.82 | Inaudible | 60 | 84 | 85 | 84 |
| 7 | F | 40 | 62 | 1.59 | 24.52 | Audible | 53 | 48 | 48 | 50 |
| 8 | M | 30 | 80 | 1.73 | 26.73 | Inaudible | 55 | 40 | 53 | 110 |
| 9 | M | 37 | 72 | 1.72 | 24.34 | Audible | 74 | 60 | 75 | 71 |
| 10 | M | 47 | 81 | 1.78 | 25.56 | Inaudible | 52 | 41 | 63 | 50 |
| 11 | M | 43 | 88 | 1.80 | 27.16 | Audible | 60 | 42 | 53 | 63 |
| 12 | M | 39 | 77 | 1.71 | 26.33 | Inaudible | 61 | 53 | 68 | 53 |
| 13 | M | 43 | 72 | 1.72 | 24.34 | Audible | 41 | 37 | 56 | 50 |
| 14 | M | 37 | 84 | 1.73 | 28.07 | Inaudible | 58 | 43 | 58 | 68 |
| 15 | M | 34 | 80 | 1.72 | 27.04 | Audible | 65 | 50 | 71 | 69 |
| 16 | F | 39 | 49 | 1.62 | 18.67 | Inaudible | 48 | 50 | 132 | 50 |
| 17 | F | 36 | 60 | 1.63 | 22.58 | Audible | 72 | 33 | 142 | 52 |
| 18 | F | 37 | 50 | 1.60 | 19.53 | Inaudible | 41 | 47 | 39 | 54 |
| 19 | F | 34 | 59 | 1.63 | 22.21 | Audible | 51 | 43 | 60 | 60 |
| 20 | F | 28 | 72 | 1.79 | 22.47 | Inaudible | 61 | 86 | 46 | 57 |
| 21 | F | 48 | 52 | 1.50 | 23.11 | Audible | 54 | 43 | 47 | 57 |
| 22 | F | 47 | 52 | 1.54 | 21.93 | Inaudible | 41 | 58 | 50 | 70 |
| 23 | F | 39 | 60 | 1.62 | 22.86 | Audible | 60 | 48 | 75 | 43 |
| 24 | F | 36 | 48 | 1.70 | 16.61 | Inaudible | 39 | 52 | 53 | 74 |
| 25 | F | 35 | 85 | 1.63 | 31.99 | Audible | 58 | 88 | 56 | 48 |
| 26 | M | 33 | 74 | 1.78 | 23.36 | Inaudible | 40 | 40 | 91 | 74 |
